# Adolescent Young Carers Who Provide Care to Siblings

**DOI:** 10.3390/healthcare12030316

**Published:** 2024-01-25

**Authors:** Rosita Brolin, Elizabeth Hanson, Lennart Magnusson, Feylyn Lewis, Tom Parkhouse, Valentina Hlebec, Sara Santini, Renske Hoefman, Agnes Leu, Saul Becker

**Affiliations:** 1 Department Health and Caring Sciences, Linnaeus University, SE-39182 Kalmar, Sweden; lennart.magnusson@lnu.se; 2 The Swedish Family Care Competence Centre, Region Kalmar, Strömgatan 13, SE-39232 Kalmar, Sweden; 3 School of Nursing 179, Vanderbilt University School of Nursing, 461 21st Avenue South, Nashville, TN 37240, USA; feylyn.m.lewis@vanderbilt.edu; 4 School of Psychology, University of Sussex, Falmer, Brighton BN1 9QQ, UK; tp93@sussex.ac.uk; 5 Faculty of Social Sciences, University of Ljubljana, 1000 Ljubljana, Slovenia; valentina.hlebec@fdv.uni-lj.si; 6 Centre for Socio-Economic Research on Ageing, IRCCS INRCA—National Institute of Health and Science on Ageing, 60124 Ancona, Italy; s.santini2@inrca.it; 7 The Netherlands Institute for Social Research (SCP), Postbus 16164, 2500 BD The Hague, The Netherlands; r.hoefman@scp.nl; 8 Institute for Biomedical Ethics, Science and Medical Faculty, University of Basel, 4001 Basel, Switzerland; agnes.leu@unibas.ch; 9 Faculty of Health and Education, Manchester Metropolitan University, Manchester M15 6BX, UK; s.becker@mmu.ac.uk

**Keywords:** sibling carer, young carer, school outcomes, mental health, well-being, quality of life, support

## Abstract

A child’s disability, long-term illness, or mental ill-health is known to affect siblings’ health, social life, school engagement, and quality of life. This article addresses a research gap by its focus on young sibling carers and the impact of providing care to a sibling. A cross-national survey study was conducted in 2018–2019 (Italy, the Netherlands, Slovenia, Sweden, Switzerland, the UK) to examine the incidence of adolescent sibling carers, the extent of care they provide, and their self-reported health, well-being, and school situation. The survey was completed by 7146 adolescents, aged 15–17, and 1444 of them provided care to family members with health-related conditions. Out of these, 286 were identified as Sibling Carers and 668 as Parent Carers, while 181 had both sibling(s) and parent(s) with health-related conditions, and thus were identified as Sibling–Parent Carers. Sibling Carers and Sibling–Parent Carers carried out higher levels of caring activities compared to Parent Carers. They reported both positive aspects of caring, such as increased maturity, and negative aspects, such as mental ill-health, impact on schooling and a lack of support. To reduce the negative aspects of a sibling carer role, it is important to recognise them and to implement early preventive measures and formal support.

## 1. Introduction

A child’s long-term illness, disability, mental ill-health, or other condition that entails an increased need for care affects not only the child and their parents but also their siblings. It affects the siblings’ home life, their interactions at school, and their interactions with friends [1]. Some of these siblings can be identified as young carers (YCs) as they provide care to the ill or disabled child [2]. YCs are defined in the literature as ”children and young people under 18 who provide or intend to provide care, assistance or support to another family member. They carry out, often on a regular basis, significant or substantial caring tasks and assume a level of responsibility which would usually be associated with an adult” [3] (p. 378). Their care recipient is often a parent or another adult who is close to them, but can also be, for example, a sibling, a friend, or another person close to them [2]. Previous research in the YC field has focused on YCs in general, i.e., YCs of all the above-mentioned categories [2] or only on selected ones [4,5], while research with a specific focus on children and adolescents who provide care to an ill or disabled sibling (so-called young Sibling Carers) is very limited.

There are a number of studies that have focused on siblings of chronically ill or disabled children, but it is not clear whether these siblings provide/d care to their ill or disabled sibling, and if so, the extent of their caring role (see, for example, [1,6,7]). However, studies have shown that siblings of chronically ill or disabled children often feel worried [1,8,9,10] and overly responsible for their sibling [1]. They often admit a protective and advocacy role [9], positioning themselves as adults within the family, taking on parental responsibilities [11], adopting the role of a ‘social glue’ in key relationships, setting their own needs aside, and distinguishing their home life from school life [12]. Their participation in everyday life is often carried out with their siblings in mind and with the family needs at the forefront [13], at the cost of their school engagement [14]. They often experience a conflict between school and being with their sibling [9] and strive to maintain a balance between their sibling’s illness and needs, their family organization, and their own needs [7,15]. Their physical health, psychosocial health, and total health scores have been found to be lower [16], with more cognitive, emotional, social, and motor difficulties [17], as well as more behavioural and psychosomatic symptoms [18] compared to their peers. Children who have a sibling with a life-threatening condition had higher rates of health care encounters, diagnoses, and medication prescriptions compared to children who did not have a sibling with a condition [6]. High rates of traumatic stress symptoms were found among siblings of children with cancer [19]. Furthermore, previous research showed low parental awareness of the extent to which the ill or disabled child’s condition affects the siblings physically and psychosocially [16], and parents are not always aware of the siblings’ symptoms of depression or anxiety [1].

Research on the impact on siblings’ quality of life (QoL) shows varying results. Most studies show lower QoL among children who have a sibling with a chronic or life-threatening illness compared to children with healthy siblings [17,20]. Siblings of children who have survived leukaemia had better QoL self-perceptions for self-esteem and psychological wellbeing, but lower QoL self-perceptions for relationships with friends and leisure dimensions compared to the reference population [21]. On the other hand, siblings of children with cystic fibrosis and siblings of children with severe motor and intellectual disabilities showed higher QoL scores compared to the general population of children of the same age [22,23].

Siblings of children with cancer reported increased popularity amongst peers [11] who frequently asked them about the ill child [10,11]. On the other hand, they stated that they were bothered by all these questions [10], that they easily get angry in school and with their schoolmates [24], that the support from their peers was inconsistent, and their contact with friends was reduced [11]. They scored lower in social relationships [25] and were found to have reduced levels of participating in social activities [10,25,26]. Furthermore, siblings of children with cancer or other conditions with complex needs were found to have less engagement in school activities [14,27] and decreased levels of school attendance [11,14,28] and school performance [10,11,25].

Research has also revealed some positive impacts of being a sibling of a child with a health-related condition. For example, greater maturity [1,26] and compassion [1], development of positive psychosocial traits, including elevated empathy and compassion [29], and a close relationship with the family and the disabled or ill sibling [9,13,23,26]. Furthermore, previous studies have shown that siblings of children with cancer receive social support primarily from friends, secondarily from family members, and thirdly from teachers [30]. They valued support from peers and teachers [26,27].

In summary, the research concerning siblings of children with ill health and/or a disability or substance abuse highlights the overall negative impacts, but also shows some positive aspects. Yet, the research does not tend to directly focus on or highlight the siblings’ caring roles.

A cross-national analysis of six European countries’ legislations found a lack of legislations that make specific reference to, or specifically recognise, children’s caring roles [31]. Children as carers were only recognised in the four countries in the United Kingdom (UK) (England, Wales, Scotland, and Northern Ireland), but young Sibling Carers are not specifically mentioned in these national legislations. However, the Carers (Scotland) Act [32] recognises YCs of families, friends, and neighbours, which means that young Sibling Carers are included, while the Children (Northern Ireland) Order [33] only includes children who provide care to a person aged 18 years or more. Swedish legislation does not recognise children as caregivers. However, the Swedish Health Care Act [34] stipulates that children should be given special consideration if their parent has a mental disorder or disability, a serious physical illness or injury, is addicted to alcohol or other addictive agents, or unexpectedly dies. Thus, young Sibling Carers are not recognised in the Swedish Health Care Act. Legislations in Italy, the Netherlands, Slovenia, and Switzerland do not make any specific references to, or specifically recognise, children as caregivers [31].

This study forms part of a larger cross-national study conducted within the major EU Horizon-funded research and innovation project, Psychosocial support for promoting Mental health and Well-being among adolescent young carers in Europe—ME-WE [35]. As all children, according to UNCRC article 12 [36], have the right to express their views and be listened to, and no policy regarding children should be designed without their voices being heard [37], the project assumed that adolescents are experts on their own unique life situation. Thus, 15–17-year-old adolescents in six European countries—Italy, the Netherlands, Slovenia, Sweden, Switzerland, and the UK—were asked to complete an online survey, with the aim of gaining insight into the profiles, caring activities, needs and preferences of Adolescent Young Carers (AYCs). The cross-national survey generated a large dataset; based on this same dataset, three previous studies have been carried out with different focuses: AYCs in general [2], AYCs who provide care to grandparents [5], and AYCs who provide help and support to friends [38].

The present study focuses on AYCs who provide care, help, or support to a sibling who has a disability or suffers from a long-term illness, mental ill-health, or addiction. These AYCs are called Sibling Carers in this paper. Comparisons are made between AYCs and adolescents who do not provide care to persons with a health-related condition; such people are called non-carer peers in this paper. Comparisons are also made between Sibling Carers and non-carer peers, as well as between Sibling Carers and adolescents who provide care to a parent, who, in this paper, are called Parent Carers.

The aim of this study was to gain increased knowledge about Sibling Carers in six European countries, the extent of the care they provide, how this affects their health, well-being, and school outcomes, as well as their current access to support. A further aim was to compare their situation with the situation of Parent Carers.

## 2. Materials and Methods

Data collection was carried out using the questionnaire which was created for the first online survey in the ME-WE project and was “made available in two data collection periods: April 2018–December 2018 (all six countries) and January 2019–July 2019 (the Netherlands, Sweden, Switzerland, and the UK only)” [2]. The questionnaire started with demographic questions about age and gender (including gender identity), family composition, place of residence, and nationality/citizenship, followed by three validated instruments with high reliability: The Multidimensional Assessment of Caring Activities (MACA-YC18) [39]; Kidscreen-10 [40]; and Positive and Negative Outcomes of Caring (PANOC-YC20) [39]. The MACA-YC18 includes 18 items rated on a three-point scale, from “never” = 0, to “some of the time” = 1, and “a lot of time” = 2, with a possible total score range of 0 to 36. A total MACA score of 0 indicates no caring activities, while scores of 10–13 indicate a moderate amount of caring activities, 14–17 a high amount, and 18 and above indicate a very high amount of caring activities [41]. The Kidscreen-10 includes ten items about health-related quality of life, with a possible total score range of 10–50; higher scores indicate greater well-being [40]. The PANOC-YC20 includes 20 items about positive and negative outcomes of care provision. Each item is rated from “never” = 0, to “some of the time” = 1, and “a lot of time” = 2. The PANOC-YC20 contains two subscales, one with ten items about positive outcomes, and one with ten items about negative outcomes of care provision. The potential range is from 0 to 20 on both subscales, with higher scores indicating a greater positive and negative score, respectively. Scores less than 12 on the positive scale and/or greater than 8 on the negative scale indicate potential concern [41].

The questionnaire also included questions about education, employment, self-rated general health status, as well as about their current access to formal and informal support [2]. Sibling Carers and Parent Carers were identified by their responses to four questions about (1) having family members with a health-related condition; (2) what kind of health-related condition (physical disabilities, mental illness, cognitive impairments, addiction, or other health-related conditions); (3) their relation to each of these family members (e.g., mother, father, stepmother, stepfather, sister, brother, grandmother, grandfather, etc.); (4) if they provide care, help, or support to any of these family members and, if so, to how many family members.

The survey was published on a web page and the 1ka platform was used to guarantee participants’ anonymity and privacy. After selecting their language on the web page, participants received a description of the study, followed by a link to the survey. The survey was designed to fit different types of electronic devices. In Italy, and in exceptional cases in the other five countries, a paper questionnaire was used due to the limited availability of electronic devices. Country partner research teams then transferred the paper data to the online platform and checked for data entry accuracy. The time for completing the questionnaire was about 30 min maximum.

Two inclusion criteria were used: being 15–17 years old and available to fill in the questionnaire. The strategy for all country partners was to recruit participants from urbanized as well as from less urbanized and rural areas, and to use various recruitment channels, such as schools, care organizations, care recipients’ interest groups, and municipalities [2]. However, during the recruitment phase, it turned out that it was not always possible to fully follow the original recruitment strategy. It sometimes was difficult to reach rural areas. Sweden mainly recruited in schools in one county, while Italy mainly recruited in high schools of two regions. Slovenia and Switzerland recruited in vocational schools—only in the German-speaking part of Switzerland—while the Netherlands recruited in schools, patient and carer organisations, care support centres, and social media channels. Finally, the UK mainly used formal support organisations for young carers, such as young carers’ festivals, social media, and a small number of schools, for their recruitment [2].

Data collection was carried out at the schools, during class time. All pupils in the classes were then invited to fill in the questionnaire. However, some of the respondents in the Netherlands and the UK completed the survey during their leisure time.

The IBM SPSS Statistics (version 25.0, Armonk, NY, USA: IBM Corp.) was used for the analysis of data. For the demographic parts, descriptive statistics, including frequency, mean and standard deviation, were used. To compare findings in different groups, descriptive and inferential statistics, including independent samples *t*-tests, were used.

Ethical approval or detailed ethics opinions (Switzerland) were secured by all country partners in their respective countries. In all cases, respondents were recruited on a voluntary basis in accordance with international declarations, regulations, and guidance documents [42,43,44]. To ensure participants’ anonymity, no registration was required to access the survey.

The first page of the survey showed an information sheet and a consent form for participants to approve. Both documents were written in clear, easily understandable language, adapted to participants’ age. The information sheet described all relevant aspects of the research protocol, foreseen benefits, possible risks of participation, that their participation was anonymous, and that they had the right to withdraw at any time if they wanted, without this leading to any consequences. Furthermore, country-specific referral mechanisms were added, including external education, care, and support professionals in case of need for this. Once they had submitted their informed consent, participants could proceed to the questionnaire. In cases where a paper questionnaire was used, participants first received an information sheet on paper and a consent form to sign. The signed consent forms were then collected by a research team member who then gave them the paper questionnaire. Where necessary due to national legislation, informed consent was also secured from the participants’ legal guardians. In addition to national laws and EU laws, each country partner followed the General Data Protection Regulations (GDPRs) [45].

A university-hosted provider was used to host the survey to assure appropriate security and data protection. Each completed questionnaire was designated an identity number, and no data were collected that could potentially identify any individual respondents. Collected online data were carefully stored and encrypted in keeping with the University of Sussex’s information security policies (https://www.sussex.ac.uk/infosec/policies accessed on 9 November 2023). All paper documents were stored in a locked filing cabinet at the partner research institution. Only authorized members of the respective national research team had access to the data.

## 3. Results

### 3.1. Participant Characteristics

The number of adolescents (15–17 years) who completed the survey was 7146, of which 60.1% were female, 38.3% male, and 1.6% non-binary, transgender, or other gender. Most of them (90.6%) were born in the country where the survey took place, and 64.5% lived in urban areas.

The total number of identified AYCs was 2099. Out of those AYCs, 286 (13.6%) had at least one sibling, but no parent or stepparent, with a health-related condition, and thus were identified as Sibling Carers, while 668 (31.8%) had at least one parent or stepparent, but no sibling, with a health-related condition, and thus were identified as Parent Carers. Finally, 181 (8.6%) were identified as Sibling–Parent Carers, as they had both siblings and parents with health-related conditions. In addition, some of the AYCs in all three groups also had a grandparent, aunt, uncle, cousin, or other family member with a health-related condition. This applies to 35 (12.2%) of the 286 Sibling Carers, 114 (17.1%) of the 668 Parent Carers, and 33 (18.2%) of the 181 Sibling–Parent Carers. The majority in all three groups were female (Sibling Carers 74.8%, Parent Carers 70.5%, Sibling–Parent Carers 82.3%). The highest proportions of Sibling Carers, Parent Carers, and Sibling–Parent Carers were found in the UK, followed by the Netherlands (Table 1).

### 3.2. The Care-Recipient Siblings’ Health-Related Condition

To find out the prevalence of different health-related conditions among the care-recipient siblings, we had to consider that 35 of the Sibling Carers also had other family members, e.g., grandparents, aunts, uncles, or cousins, with health-related conditions. To make sure that the results of the frequency analysis only show the health-related condition of the care-recipient siblings, and not that of other family members, we excluded the responses from those 35 Sibling Carers. Thus, out of the 286 Sibling Carers, only 251 were included in this analysis. The most common health-related condition among the care-recipient siblings was cognitive impairment (44.2%), followed by mental ill-health (35.9%), physical disabilities (24.7%), and other health conditions (21.5%) such as allergies, cancer, diabetes, epilepsy, and heart problems, while addiction only occurred in a few cases (2.4%).

### 3.3. The Extent of Caring Tasks and the Effects of Caring Responsibilities

Independent samples *t*-tests of the MACA-YC18 results showed that compared to their non-carer peers (M = 8.81, SD = 4.57), AYCs perform greater amounts of caring activities (M = 12.57, SD = 5.64), *t* (3210.93) = 26.73, *p* < 0.001, d = 0.73, with a mean value of 14.24 (SD = 6.14) for Sibling Carers, compared to 12.51 (SD = 5.40) for Parent Carers (*t* = 4.082; *df* = 475.716; *p* < 0.001 (equal variances not assumed)), and 14.80 (SD = 6.07) for Sibling–Parent Carers (*t* = −0.955; *df* = 457; *p* = 0.340).

The PANOC-YC20 was used to assess positive and negative effects of the caring role, with scores below 12 on the PANOC positive scale and/or scores above 8 on the negative scale indicating potential concern (Table 2).

The Sibling Carers’ results on the positive scale (M = 12.86; SD = 4.66), as well as on the negative scale (M = 5.53; SD = 5.14), were similar to those of Parent Carers (M = 12.44; SD = 4.69, and M = 5.68; SD = 5.10). However, when comparing the PANOC results of Sibling Carers with those of Sibling–Parent Carers, the latter group had lower scores on the positive scale (M = 11.62; SD = 4.67) (*t* = 2.564; *df* = 384; *p* = 0.011) and significantly higher scores on the negative scale (M = 8.15; SD = 5.75) (*t* = −4.688; *df* = 307.654; *p* < 0.001 (equal variances not assumed)).

### 3.4. AYCs’ Health Condition and Quality of Life

The participants were asked to rate their general health status on a 5-point scale, in which higher scores indicated poorer health: 1 = ‘Excellent’; 2 = ‘Very good’; 3 = ‘Good’; 4 = ‘Fair’; 5 = ‘Poor’. Independent samples *t*-tests showed that the self-rated general health status was significantly poorer among Sibling Carers (M = 2.78; SD = 1.03) compared to their non-carer peers (M = 2.48; SD = 1.05) (*t* = −4.732; *df* = 300.896; *p* < 0.001 (equal variances not assumed)), but not as poor as among the Parent Carers (M = 3.06; SD = 1.12) (*t* = −3.561; *df* = 914; *p* < 0.001). The poorest self-rated health status was found among the Sibling–Parent Carers (M = 3.47; SD = 1.06); their reported health status was significantly poorer compared to the Parent Carers (M = 3.06; SD = 1.12) (*t* = −4.405; *df* = 817; *p* < 0.001). Mental health problems were the most common health-related conditions among the AYCs, with the highest incidence among Sibling–Parent Carers (57.4%) followed by Parent Carers (39.3%) and Sibling Carers (34.8%). The second most common health-related condition was learning difficulties, with the highest incidence among Sibling–Parent Carers (16.5%), followed by Parent Carers (12.4%), and Sibling Carers (8.3%). Other self-reported health-related conditions were, for example, ADHD, dyslexia, physical disabilities, and autism spectrum disorders.

The Kidscreen−10 instrument was used to gain knowledge about the adolescents’ health-related quality of life (QoL). A total score of 50 indicates an extremely high level of health-related QoL. To investigate possible differences between the groups, a series of independent samples *t*-tests were used. The results showed significantly lower scores for Sibling Carers (M = 33.79; SD = 7.89) compared to their non-carer peers (M = 36.21; SD = 6.71) (*t* = 4.942; *df* = 285.546; *p* < 0.001 (equal variances not assumed)), but slightly higher scores compared to Parent Carers (M = 32.76; SD = 7.46) (*t* = 1.859; *df* = 890; *p* = 0.063). The lowest level of health-related QoL was found in the Sibling–Parent Carers group, with a mean value of 29.94 (SD = 7.53), which was significantly lower compared to the Sibling Carers (*t* = 5.104; *df* = 441; *p* < 0.001) and compared to Parent Carers (*t* = 4.399; *df* = 797; *p* < 0.001).

### 3.5. Difficulties Due to the Caring Role

Some of the AYCs in all three groups reported that their caring role had a negative impact on their school situation, and that they had been bullied because of caring. Both these impacts of the caring role were most common in the Sibling–Parent Carers group followed by the Sibling Carers group (Table 3). In some cases, the caring role led to thoughts of hurting oneself, the care recipient, or someone else. Thoughts of hurting oneself or the care recipient were most common in the Sibling–Parent Carers group, while thoughts of hurting someone else were most common in the Sibling Carers group (Table 3).

### 3.6. AYCs’ Access to Support

Finally, the AYCs were asked about their access to formal and informal support. The results showed that more than half of the AYCs did not have access to any kind of formal support, yet more than half of them reported informal support from friends. Parent Carers reported the least access to formal support in connection to their caring role, and the least access to formal family support, as well as the least access to support in school (Table 4).

## 4. Discussion

The aim of this study was to gain increased knowledge about adolescent Sibling Carers in six European countries, the extent of the care they provide and how this affected their health, well-being, and school outcomes, as well as their current access to support. A further aim was to compare their situation with the situation of adolescent Parent Carers.

The results show that Sibling Carers performed greater amounts of caring activities than Parent Carers. The reasons for this difference cannot be deduced from this study and therefore need to be further investigated. The Sibling–Parent Carers group performed the greatest amount of caring activities, which is not surprising, since the Sibling–Parent Carers’ families include at least one child and one adult who have a health-related condition.

The impacts of a sibling-caring role highlighted some positive aspects, which confirms previous research on being a sibling to a child with a chronic/life-threatening illness or physical/cognitive disability [1,13,23,29]. On the other hand, the results also showed negative aspects of the sibling-caring role. Negative aspects have also been highlighted in previous research on being a sibling to a child with a chronic/life-threatening illness [1,6,13,16,20]. In our study with an explicit focus on the caring roles of siblings, the PANOC results indicated potential concern for 29.4% of the Sibling Carers who scored below 12 on the positive scale, and for 21.3% of the Sibling Carers who scored above 8 on the negative scale. These figures were higher among Parent Carers (34.3% and 22.0%) and highest among Sibling–Parent Carers (42.0% and 41.4%). In summary, the findings indicate that there are many adolescent Sibling Carers, Parent Carers, and Sibling–Parent Carers who are in need of additional help and support.

The Sibling Carers’ self-rated health status was poorer than among their non-carer peers. This result is in line with previous research on siblings to children with chronic/life-threatening illnesses [6,16]. Despite the Sibling Carers’ higher levels of caring activities, their health status was not as poor as among Parent Carers or Sibling–Parent Carers. This could be due to Sibling Carers having parents who are able to provide them with some support. It could also be due to Sibling Carers having access to formal support to a greater extent than Parent Carers.

The most common health problem in all three groups was mental ill-health, which confirms previous research findings of poorer psychosocial health [16], more emotional difficulties [17], more behavioural and psychosomatic symptoms [18], and higher rates of traumatic stress symptoms [19] among siblings of children with chronic/life-threatening illnesses.

The Sibling Carers’ self-reported health-related QoL was lower compared to their non-carer peers, which confirms previous research on siblings of children with a chronic/life-threatening illness [17,20] and contradicts previous research on siblings of children with severe motor and intellectual disabilities [23]. Sibling Carers’ health-related QoL was almost as low as among Parent Carers and Sibling–Parent Carers, who scored lowest. Furthermore, Sibling Carers reported school difficulties, and negative impacts on school results, to a greater extent compared to Parent Carers. Negative impact on schooling has also been shown in research on siblings of children with cancer or other conditions with complex needs [10,11,14,25,27,28]. Sibling Carers also experienced bullying because of their caring role to a greater extent than Parent Carers.

More than 40 percent of the Sibling Carers and Sibling–Parent Carers received formal support, which can be considered relatively high in comparison to that received by Parent Carers (roughly 34 percent). Further research is needed to find out the causes of these differences. Nevertheless, this means that the majority of the AYCs in all three groups did not have access to any kind of formal support, and for the majority, no one in school was aware of their caring role. These findings are in line with a previous study in which young people who provided care to family members reported high levels of need but low levels of support [4]. However, while missing formal support, more than half of the AYCs in all three groups had a close friend who knew about their caring role, which is consistent with previous studies showing that adolescents often prefer to confide in and seek support from friends rather than from adults [46,47,48]. On the other hand, young people who provide care to family members have requested greater support for their care recipients [4], and the ‘Young Carers projects’ in England have been evidenced to help reduce stress among young people who provide care to family members and encourage them to relax [49].

According to the United Nations Global Sustainability Development Goals (UNSDGs) [50], every society should strive to ensure healthy lives and promote well-being and lifelong learning opportunities for all. It is every society’s responsibility to ensure that young people’s caring responsibilities do not become too onerous, interfere with their education, or become harmful to their health and development [36] (article 32). Our study results indicate that, when it comes to adolescent Sibling Carers in the six European countries, much remains to be done to fulfil the above-mentioned UNSDGs and the UNCRC article 32. The Sibling Carers’ self-reported negative impact on their health, wellbeing, and school situation, as well as their experiences of bullying, and the fact that some of them had thoughts about hurting themselves or someone else, are worrying results that demonstrate high levels of need for support while, at the same time, most of them had no access to formal support.

It could be argued that minors should not have these caring responsibilities, and that the formal services should take over the care. Yet, our results, as well as previous research [51], indicate that this is not the case today. To achieve a more sustainable future for young Sibling Carers, it is important that they are recognised and, further, that all young Sibling Carers are supported. This could be achieved, for example, by having national legislations that recognise children and adolescents as informal carers, not only to parents or other adult family members, but also to siblings under the age of 18, and to have support services in place for carers under the age of 18.

Specific attention should be paid to Sibling–Parent Carers, who seem to be the most vulnerable group of the young Sibling Carers in our study. Recognition in national legislations would contribute to raised awareness of young Sibling Carers and increased possibilities to identify them, to give them the opportunity to express their views and needs, and to offer them the support they need. Their voices need to be heard to make sure that the offered support is truly based on their experiences, needs, and preferences, as was carried out in a co-designed intervention for AYCs, which was developed and tested in the ME-WE AYC project [35], with the goal to promote their mental health and wellbeing [52]. Furthermore, there are examples of good practices which are aimed to support YCs in general and implemented within the framework of non-specific legislation, for example, support in school and/or from child and adolescent mental health teams, as well as peer support groups, summer camps, and online platforms for YCs [51], which can serve as support for young Sibling Carers. However, since their situation differs from that of young Parent Carers, Sibling Carers need to be better identified and, in addition to support aimed at YCs in general, it is important they are also offered more specific, tailored support, such as support groups and interventions for young Sibling Carers. In addition to direct support for young Sibling Carers, it is important to provide formal support to their parents since this can act as indirect support for the young Sibling Carers.

### Strengths and Limitations of the Study

This study provides, for the first-time, demographic information on adolescent Sibling Carers at a European level, investigating their self-reported health, wellbeing, school outcomes, and the support they receive. By using validated instruments (MACA-YC18, PANOC-YC20, Kidscreen-10), the study provides knowledge about Sibling Carers’ caring activities, positive and negative outcomes of their caring role, and the caring role’s impact on their health, wellbeing, and school situation. Furthermore, the study provides new knowledge about similarities and differences when comparing the situation of adolescent Sibling Carers with the situation of adolescent Parent Carers. To achieve increased knowledge and understanding of these differences, we recommend future research to focus on explaining the differences, for example, by using multivariate analyses.

However, there are some limitations to this study. The recruitment process was associated with difficulties and obstacles which, in some countries, led to deviations from the original recruitment strategy. Due to the differences between the six countries’ sampling strategies, the samples cannot be seen as statistically representative for the population. This means that it is not possible to present a generalisable country distribution of adolescent Sibling Carers and Parent Carers. It also means that the comparisons of the outcomes among AYCs and their non-carer peers are less precise. Furthermore, the small sample sizes of the subgroups ‘Sibling Carers’, ‘Parent Carers’ and ‘Sibling–Parent Carers’ also mean that the comparisons of the outcomes among these three groups are less precise. Finally, due to the large differences in sample sizes in the different countries, it was not feasible to conduct a comparative analysis of the findings based on the adolescents’ country of origin. Despite these limitations, given the lack of scientific research among AYCs, especially adolescent Sibling Carers, the study contributes original and significant insights and knowledge regarding young Sibling Carers’ situations.

## 5. Conclusions and Implications

Adolescent Sibling Carers were found to perform greater amounts of caring activities than adolescent Parent Carers. Being an adolescent Sibling Carer may bring some positive aspects, such as increased maturity and close relationships with family members, but it also often brings negative impacts on health, well-being, and quality of life to almost the same extent as for adolescent Parent Carers, while negative impacts on schooling are more common among Sibling Carers than Parent Carers. Furthermore, most of the Sibling Carers experienced a lack of formal support in their caring role. Thus, taken together, the results highlight the importance of recognising young Sibling Carers, in legislation as well as in practice, to identify them and implement early preventive measures with a focus on minimising the negative aspects and optimising the positive aspects of their caring role. Further research with a focus on young Sibling Carers is needed to gain more knowledge about this sub-group of young carers. For example, the impact of young carers’ sociodemographic characteristics, caring tasks, and responsibilities need to be studied in more detail in future studies in order to delineate more tailored interventions targeting these sub-groups of young carers.

## Figures and Tables

**Table 1:** The proportion of Sibling Carers, Parent Carers, and Sibling–Parent Carers among the AYCs in each partner country.

	AYCs	Sibling Carers		Parent Carers		Sibling–Parent Carers	
	Total N	N	(%) *	N	(%)	N	(%)
The UK	402	83	(20.6)	163	(40.5)	61	(15.2)
The Netherlands	199	36	(18.1)	77	(38.7)	26	(13.1)
Switzerland	240	31	(12.9)	62	(25.8)	12	(5.0)
Sweden	702	84	(12.0)	223	(31.8)	59	(8.4)
Slovenia	342	33	(9.6)	114	(33.3)	22	(6.4)
Italy	214	19	(8.9)	29	(13.6)	1	(0.5)
Total	2099	286	(13.6)	668	(31.8)	181	(8.6)

* Descending order.

**Table 2:** Number of Sibling Carers, Parent Carers, and Sibling–Parent Carers scoring below 12 on the PANOC positive scale and above 8 on the negative scale.

	PANOC Positive Score below 12		PANOC Negative Score above 8	
	N	(%)	N	(%)
Sibling Carers (N = 286)	84	(29.4)	61	(21.3)
Parent Carers (N = 668)	229	(34.3)	147	(22.0)
Sibling–Parent Carers (N = 181)	76	(42.0)	75	(41.4)
Total (N = 1135)	389	(34.3)	337	(29.7)

**Table 3:** Experienced issues and difficulties due to the caring role.

	Sibling Carers (N = 286)		Parent Carers (N = 668)		Sibling–Parent Carers (N = 181)	
	N	(%)	N	(%)	N	(%)
Find school difficult because of caring	59	(21.6)	113	(17.9)	63	(36.2)
Caring has a negative impact on school results	56	(20.6)	114	(18.2)	65	(36.7)
Been bullied because of caring	62	(22.8)	100	(15.9)	57	(32.4)
Considered hurting oneself	32	(11.8)	93	(14.8)	63	(35.8)
Considered hurting others	14	(5.2)	37	(5.9)	19	(10.7)
Of those who had considered hurting others:						
The care recipient	4	(28.6)	17	(48.6)	12	(66.7)
Someone else	10	(71.4)	18	(51.4)	6	(33.3)

Note. The valid percentage is presented, ignoring missing values. For the ‘Of those who had considered hurting others’ columns, the percentage reflects the valid percentage of the participants who indicated they had considered hurting others.

**Table 4:** AYCs’ current access to formal and informal support.

	Sibling Carers (N = 286)		Parent Carers (N = 668)		Sibling–Parent Carers (N = 181)	
	N	(%)	N	(%)	N	(%)
Formal support in connection to the caring role	111	(42.7)	211	(34.3)	71	(40.8)
The family receives formal support	119	(44.9)	166	(27.2)	64	(37.6)
School knows about the caring role	107	(40.8)	213	(34.5)	81	(46.6)
A close friend knows about the caring role	165	(62.5)	354	(57.5)	115	(66.1)

Note. The valid percentage is presented, ignoring missing values.

## Data Availability

The data that support the findings of this study are available on request from the corresponding author. The data are not publicly available due to privacy and ethical restrictions.
